# Omics Science and Social Aspects in Detecting Biomarkers for Diagnosis, Risk Prediction, and Outcomes of Carotid Stenosis

**DOI:** 10.3390/biom14080972

**Published:** 2024-08-08

**Authors:** Davide Costa, Enrica Scalise, Nicola Ielapi, Umberto Marcello Bracale, Teresa Faga, Ashour Michael, Michele Andreucci, Raffaele Serra

**Affiliations:** 1 Department of Medical and Surgical Sciences, Magna Graecia University of Catanzaro, 88100 Catanzaro, Italy; davide.costa@unicz.it (D.C.); enrica.scalise@unicz.it (E.S.); 2 Interuniversity Center of Phlebolymphology (CIFL), “Magna Graecia” University, 88100 Catanzaro, Italy; 3 Department of Public Health and Infectious Disease, “Sapienza” University of Rome, 00185 Roma, Italy; nicola.ielapi@uniroma1.it; 4 Department of Public Health, University Federico II of Naples, 80131 Naples, Italy; umbertomarcello.bracale@unina.it; 5 Department of Health Sciences, Magna Graecia University of Catanzaro, 88100 Catanzaro, Italy; teresa_faga@yahoo.it (T.F.); ashourmichael@yahoo.com (A.M.)

**Keywords:** carotid artery stenosis, prevention, progression, omics, genomics, transcriptomics, proteomics, biomarkers and social determinants of health

## Abstract

Carotid stenosis is characterized by the progressive narrowing of the carotid arteries due to the formation of atherosclerotic plaque, which can lead to stroke and death as major complications. Numerous biomarkers allow for its study and characterization, particularly those related to “omics” sciences. Through the most common research databases, we report representative studies about carotid stenosis biomarkers based on genomics, transcriptomics, proteomics, and metabolomics in a narrative review. To establish a priority among studies based on their internal validity, we used a quality assessment tool, the Scale for the Assessment of Narrative Review Articles (SANRA). Genes, transcriptomes, proteins, and metabolites can diagnose the disease, define plaque connotations, predict consequences after revascularization interventions, and associate carotid stenosis with other patient comorbidities. It also emerged that many aspects determining the patient’s psychological and social sphere are implicated in carotid disease. In conclusion, when taking the multidisciplinary approach that combines human sciences with biological sciences, it is possible to comprehensively define a patient’s health and thus improve their clinical management through precision medicine.

## 1. Introduction

Carotid artery stenosis (CS) is an important cause of stroke and the fifth-leading cause of death in Western countries. The incidence of CS varies across populations, but it is generally around 5–7% of the general adult population aged 50 years or older, with the incidence increasing linearly with age. Risk factors for CS include those typically associated with atherosclerotic vascular disease, including age, male gender at birth, smoking, hypertension, dyslipidemia, and family history [1,2,3,4].

CS is a progressive disease, and affected patients should be followed with long-term imaging throughout their lives. Factors influencing the progression of carotid disease and the subsequent occurrence of its main complication, ischemic stroke, are not yet fully understood [5]. Still, precision medicine (PM) research is currently active, with several experiences published in predictive modeling [6].

Currently, the available treatments for CS are medical management with risk reduction, including the management of hypertension, diabetes, and hypercholesterolemia. Furthermore, surgical intervention is proposed for symptomatic patients with arterial stenosis greater than 50% if the patient experiences a carotid-related event such as a cerebrovascular accident or for asymptomatic disease with arterial stenosis greater than 60% if the perioperative stroke and mortality risk is <3% [7]. Treatment options consist of revascularization procedures such as carotid endarterectomy (CEA) and carotid artery stenting (CAS), which may be associated with postoperative complications, such as carotid restenosis [8,9]. Even in this latter case, PM may help physicians predict which patients are at a major risk of developing complications after treatment. In the context of PM and precision health (PH), omics sciences have recently become very popular in cardiovascular diseases [10,11,12]. In particular, omics refers to several disciplines in biomedical sciences that end with -omics, such as genomics, transcriptomics, proteomics, and metabolomics [6]. Furthermore, certain biomarkers may also represent the social issues around some cardiovascular diseases and fundamental lesions such as atherosclerosis [13]. Physicians typically focus on traditional risk factors when evaluating cardiovascular risk, but these factors provide only a limited perspective on the complexities encountered by individuals with cardiovascular disease. Essential elements like the psychosocial status and its effect on mental health are often neglected [14]. PM aims to develop personalized treatments by integrating biological factors, interindividual variability, and various health determinants [6]. Considering the significant impact of the psychosocial domain on cardiovascular diseases, precision medicine holds considerable promise for application in this research area [13,14].

This review aims to review the most updated literature on omics-based biomarkers, social aspects, and CS.

## 2. Methods

We searched Web of Science, Scopus, ScienceDirect, and Medline to find relevant articles published between 2004 and 2024 (the last two decades). The keywords used with various combinations were “carotid artery stenosis”, “prevention”, “progression”, “omics”, “genomics”, “transcriptomics”, “proteomics”, “biomarkers”, and “social determinant of health”. We selected in vivo animal and human studies concerning omics approaches (genomics, transcriptomics, proteomics, metabolomics), including additional social aspects to detect new biomarkers for diagnosis, risk prediction, and outcomes of carotid stenosis. To improve standardization and to establish a priority among studies based on their internal validity, we used a quality assessment tool, the Scale for the Assessment of Narrative Review Articles (SANRA), based on answers to six items [15]. The scale consists of six items that range as follows: 0 (low quality), 1 (intermediate quality), and 2 (high quality). It encompasses the following areas: description of (1) the significance and (2) the objectives of the article, (3) research in the existing literature and (4) citation methods, and presentation of (5) the evidence quality and (6) pertinent endpoint information [15].

## 3. Results

### 3.1. Quality Assessment of the Included Studies

Using the SANRA, the studies presented in this narrative review received a quality rating ranging from intermediate (score 1) to high (score 2) for each item, totaling a score between 12 and 10. The SANRA evaluation of the articles related to genomics, transcriptomics, proteomics, metabolomics, and social aspects is provided in the following tables (Table 1, Table 2, Table 3, Table 4 and Table 5) to give the reader a weighted analysis of the selected studies.

### 3.2. Genomics and Carotid Stenosis

Carotid stenosis (CS) has been extensively studied from a genomic perspective. This brief overview provides the recent studies focusing on genetic variations concerning the development of carotid stenosis [16,17], atherosclerotic plaque instability [18,19,20,21], other cardiovascular diseases and comorbidities [22,23,24], and the outcomes of revascularization interventions [25]. The articles are presented according to the themes mentioned above and their internal quality, with preference given to those with 12 as the total score for describing events related to genomics (development of carotid stenosis, atherosclerotic plaque instability, other cardiovascular diseases, comorbidities, and outcomes after carotid revascularization procedures) and using the articles with a total score of 10 to support the underlying mechanisms. 

Table 6 summarizes the principal features of the reviewed articles.

Cytochrome P-450 (CYP) enzyme genetic variants have been linked to carotid stenosis, and specific CYP450 gene single-nucleotide polymorphisms (SNPs) are associated with CYP450 metabolites, such as 20-hydroxyeicosatetraenoic acid, total epoxyeicosatrienoic acids, and dihydroxyeicosatrienoic acids, which may contribute to the development of carotid stenosis [16]. Additionally, bioinformatics were used to analyze certain upregulated genes linked to immune and inflammatory responses in carotid stenosis [17]. 

Carotid plaque stability is an essential determinant of carotid atherosclerotic progression, and genomics studies have investigated this issue. These studies [18,19] focused on identifying markers for the progression of carotid atherosclerosis and potential targets for its treatment to avoid rupture. Pleckstrin (PLEK) and polymorphisms of the osteoprotegerin (OPG) gene (that encodes for the secretory glycoprotein osteoprotegerin) may serve as a crucial biomarker and a potential therapeutic target correlated with monocyte and macrophage activity responsible for plaque progression [18,19]. A further study [20] identified significant upregulation of the chemokine (c-c motif) ligand 19 (CCL19) gene’s expression in clinically unstable plaques. A specific gene variant p. (Gln787=) in the gene that encodes epidermal growth factor receptor (EGFR) was inversely correlated with intraplaque hemorrhage and ischemic brain lesions in patients with unstable carotid plaques [21]. 

Several studies [22,23,24] provided explanatory examples to analyze the risk of carotid atherosclerotic plaque in cardiovascular diseases and comorbidities through a genomic approach. The relationship between carotid artery stenosis (CS) and ischemic cerebrovascular disease (ICVD) was explored by examining 100 gene variations for ICVD [22]. Differences in gene variations, including factor VII, apolipoprotein E, and two renin polymorphisms, were associated with ischemic cerebrovascular disease and CS more so compared to controls [22]. The study by Yi et al. [23] emphasizes the genetic basis of CS, highlighting interactions between specific genetic loci among variants responsible for platelet activation (TXA2R rs1131882, P2Y1 rs1371097, and GPIIIa rs2317676), which had a combined impact on symptomatic carotid stenosis in patients with ischemic stroke. Nine single-nucleotide polymorphisms (SNPs) are linked to carotid artery atheromatous, suggesting a risk for atherosclerotic diseases in individuals with diabetes mellitus and CS [24]. Regarding outcomes after carotid revascularization procedures, a variation in the inducible nitric oxide synthase polymorphism offers a defense against moderate to severe cognitive impairment (that appears in 9% to 23% of patients) one month after CEA. Moreover, this protection seems to influence cognitive functions on the same side as the operated carotid artery [25].

### 3.3. Transcriptomics and Carotid Stenosis

Transcriptomics has proven to be a valuable tool for understanding the molecular mechanisms of carotid stenosis. In particular, some examples are provided on transcriptomes involved in the inflammatory process leading to carotid stenosis [26,27], transcriptomic analysis of the characteristics of unstable plaque [28,29,30,31], outcomes of carotid interventions [32], and using miRNAs to more accurately delineate the characteristics of carotid stenosis [33,34]. The articles are presented according to the themes mentioned above and their internal quality, with preference given to those with 12 as the total score for describing events related to transcriptomics (development of carotid stenosis, atherosclerotic plaque instability, outcomes after carotid revascularization procedures, and the use of miRNAs) and utilizing the articles with a total score of 10 to support the underlying mechanisms.

Table 7 summarizes the principal features of the reviewed articles.

It was discovered, through a transcriptomic analysis, that the absence of a protein known as myeloid-hypoxia-inducible factor-1α (HIF1α) led to a decrease in the number of macrophages and smooth muscle cells in narrowed arteries and hindered the development of carotid neointima [26]. Moreover, upregulated gene proprotein convertase subtilisin/kexin type 6 (PCSK6) in symptomatic patients’ plaques was associated with inflammation and matrix degradation [27]. 

In the context of unstable/stable carotid plaque, morphological changes in vascular smooth muscle cell (VSMC) organelles, particularly in the endoplasmic reticulum (ER) whorls, could offer reliable biomarkers for atherosclerotic progression [28]. High-calcified plaques exhibit upregulation of genes associated with smooth muscle cells and extracellular matrix organization while showing downregulation of macrophage markers [29]. Single-cell RNA sequencing was applied to examine carotid artery plaques, and plaque-specific T-cell subsets and immune cells (monocytes and macrophages) showed mixed results regarding plaque vulnerability: some hade pro-inflammatory properties, and some had anti-inflammatory characteristics [30]. Transcriptional regulation may be crucial in determining the plaque phenotype, as demonstrated by a study [31] that identified 30 gene transcription factors linked to plaque instability, highlighting the role of inflammation proteases and hemoglobin metabolism [31]. Regarding the analysis of outcomes after surgical injury through a transcriptomic approach, RNA from injured and uninjured arteries revealed significant gene expression and protein level changes, suggesting new targets, such as Arginase I and Kruppel-like factors, to limit surgically induced restenosis [32].

Studies on CS-related transcriptomes have provided insights into miRNA (micro RNA) expression in stable and vulnerable carotid plaques [33,34]. Differentially expressed miRNAs were identified in vulnerable plaques, with associated target genes involved in various biological pathways such as protein phosphorylation, transcription, nitrogen compound metabolism, and signaling mechanisms [33]. MiR-214 is a candidate microRNA that regulates vascular smooth muscle cell (VSMC) angiogenesis, proliferation, and senescence under CoCl2 (phosgene)-induced hypoxia, suggesting that it may serve as a marker for vascular senescence and a potential therapeutic target for CS [34].

### 3.4. Proteomics and Carotid Stenosis

Proteomics has become a valuable approach to examining the molecular mechanisms underlying carotid stenosis. First, a brief overview of the proteins involved in carotid stenosis using a proteomic approach is given. Examples are proteins related to contractility in surgically induced carotid stenosis [32], proteins that may predict future cardiovascular death [35], proteins that differ according to gender in patients with carotid stenosis [35,36], and proteins associated with inflammatory processes [37,38]. Secondly, metalloproteinases in carotid stenosis are focused on from a proteomic point of view [39,40,41] and proteins that contribute to defining the vulnerability of atherosclerotic plaque [42,43,44]. The articles are presented according to the themes mentioned above and to their internal quality, with preference given to those with 12 as the total score for describing events related to proteomics (development of carotid stenosis and the proteins involved, metalloproteinases and carotid stenosis, atherosclerotic plaque instability and the proteins involved) and using the articles with a total score of 10 to support the underlying mechanisms.

Table 8 summarizes the principal features of the reviewed articles.

Some studies [32,33,34,35,36,37,38] have provided insights into the proteomic landscape associated with carotid stenosis. A proteomic investigation revealing time-dependent post-translational modifications and differential expression of proteins related to contractility in surgically induced carotid stenosis [32]. Additionally, a protein signature of four key proteins (calponin, protein C, serpin H1, and versican) accurately predicted future cardiovascular mortality, outperforming traditional imaging and histology in prognostic performance [35]. The research by Liang et al. [36] found that five proteins exhibited gender-specific changes, and males with plaque had notably higher ferritin levels. Interestingly, differences were also observed between male and female patients regarding the abundance of specific proteins (large aggregating proteoglycans versican and aggrecan), which inversely correlated with estradiol levels [35]. Furthermore, Porcelli et al. [37] identified carotid atherosclerotic plaque proteins involved in transportation, scavenging of harmful radicals, metabolic enzymatic activities, and structural support [37]. A biomarker panel of six urinary proteins, identified using data-independent acquisition (DIA) quantification, showed potential diagnostic value in differentiating symptomatic and asymptomatic CS patients associated with immune and inflammatory pathways [38]. 

In the context of proteomics research that involves metalloproteinases (MPs), matrix metalloproteinases-9 (MMP-9) is differentially expressed in plaques with advanced carotid stenosis of symptomatic subjects, suggesting its potential role as a biomarker of plaque instability that may, therefore, lead to severe events such as stroke and death [39]. Accordingly, a proteomics analysis revealed that tissue inhibitors of metalloproteinases (TIMPs) and disintegrin and metalloproteinases (ADAMs) regulate interactions between cells and the extracellular matrix (ECM), contributing to the formation of unstable plaques [40]. Reduced matrix metalloproteinase-10 (MMP-10) levels indicate early-stage carotid atherosclerosis; the protein profiles that predict this condition differ from those associated with intima-media thickness (IMT) development, possibly reflecting distinct pathological causes [41]. 

Regarding the proteomics approach in the search for biomarkers that can define plaque characteristics, including its stability or instability, many researchers have already addressed this issue [42,43,44]. Matrix metalloproteinases and cathepsins were more abundant in unstable plaques, while collagens and proteoglycans were more prevalent in stable plaques [42]. Different proteins are involved in critical pathways related to ferroptosis and lipid metabolism, and their expression patterns differ between stable/unstable plaques and lipid core regions [43]. Unstable plaques have reduced levels of protective enzymes, small heat shock proteins, annexin A10, and Rho-GDP dissociation inhibitor (GDI); conversely, unstable plaques show higher levels of ferritin light subunit and fibrinogen fragment D than stable plaques [44]. 

### 3.5. Metabolomics and Carotid Stenosis

Metabolomic studies offer valuable insights into the metabolic changes and potential biomarkers associated with CS, paving the way for improved diagnostic and therapeutic strategies to manage this vascular condition. The following paragraph briefly overviews the predicted biomarker metabolites involved in carotid stenosis [45,46,47,48,49]. It also provides some examples of the metabolic pathways that can help define the stability of the carotid plaque [50,51,52] and highlights the connections between carotid stenosis and microbiota through metabolomics [53,54]. The articles are presented according to the themes mentioned above and to their internal quality, with preference given to those with 12 as the total score for describing events related to metabolomics (development of carotid stenosis and the metabolites involved, atherosclerotic plaque instability and the metabolites involved, metabolomics, and microbiota) and using the articles with a total score of 10 to support the underlying mechanisms. 

Table 9 summarizes the principal features of the reviewed articles.

Homocysteine, choline, and lipids, along with traditional risk factors, may play a role in the development of CS. Modifying one’s diet to regulate homocysteine, choline, and lipids might be beneficial in preventing CS [45]. Moreover, significantly higher plasma homocysteine levels in male patients with severe CS indicate a potential metabolic marker for this condition [46]. Acylcarnitine species, amino acids, biogenic amines, and glycerophospholipids helped predict CS [47]. Furthermore, there are connections between serum cholesterol (high-density lipoprotein cholesterol and low-density lipoprotein cholesterol), the occurrence of carotid plaque, and the morphology of that plaque [48]. Non-esterified fatty acids (NEFAs) may be locally produced and contribute to the inflammation of diabetic carotid plaque neointima [49]. In carotid arteries containing plaques, metabolic compounds are responsible for atherosclerotic disease, including the cholesterol, purine, pyrimidine, and phosphatidylethanolamine-ceramide pathways. Moreover, the presence of acylcarnitines as β-oxidation intermediates suggests a disruption in fatty acid metabolism and possible mitochondrial dysfunction [50]. Metabolic phenotyping could be essential in studying the chemistry of unstable carotid plaque. Two biological pathways (the eicosanoid pathway and β-oxidation) allow one to distinguish between stable and unstable carotid plaque tissue [51]. Through lipidomics analysis, cholesterol, esters with long-chain fatty acids, and specific types of sphingomyelin were found to be significantly more abundant in atherosclerotic plaques of subjects who underwent CEA compared to the bloodstream. These compounds played a role in identifying lipid patterns in vulnerable carotid plaques [52]. 

Interesting studies proposed a link between microbiota and CS through metabolomics [53,54]. Wang et al. [53], in women with HIV, found a connection between certain types of bacteria in the gut, a plasma microbial metabolite called imidazole-propionate (ImP) responsible for carotid plaque build-up, and the development of carotid artery atherosclerosis [53]. Cason et al. [54] showed that plasma metabolites modulated by the microbiome, like indole, tryptophan, indole-3-propionic acid, and indole-3-aldehyde, were negatively associated with advanced carotid atherosclerosis.

These findings suggest an association between the body’s immune response and inflammation [53,54]. Some other metabolites, such as the hippuric acid kynurenine/tryptophan ratio, were linked with postoperative cardiac events in patients with advanced carotid atherosclerosis [54].

### 3.6. Social Issues and Carotid Stenosis

Several studies also considered the social issues surrounding carotid stenosis. This section provides a brief overview of the social aspects involved in carotid stenosis, which can also be regarded as biomarkers of the disease, as they are responsible for the psychological well-being of patients [55,56,57,58]. The articles are presented according to the themes mentioned above and their internal quality, with preference given to those with a total score of 12 describing social aspects of carotid stenosis. The articles with a total score 10 are used to support the underlying mechanisms. 

Table 10 summarizes the principal features of the reviewed articles.

Gender, race, and ethnicity are associated with the rates of carotid intervention during the initial hospitalization, even after comprehensive risk adjustment for clinical, social, and demographic aspects. There is a need to focus on mitigating these inequalities [55,56]. Inequalities also manifest themselves in several psychosocial aspects: individuals from neighborhoods with higher levels of social disadvantage presented more severe CS and were more inclined to undergo transfemoral carotid artery stenting [55,56,57,58]. These patients also experienced higher rates of mortality and stroke, were less likely to be discharged home, and had more extended hospital stays [55]. Accordingly, low socioeconomic status (SES) predicts postoperative mortality in CEA patients but not for CAS patients. Furthermore, CEA is associated with a higher incidence of stroke in low-SES patients [56]. It is crucial to prioritize efforts to ensure that patients with a low socioeconomic status and social disparities receive proper carotid surveillance and appropriate treatment for their disease [57]. Finally, to identify markers of health-related quality of life (HRQoL) in patients with carotid stenosis, Aber et al. [58] determined the patient-reported outcome measures (PROMs) that cover the most relevant social themes characterizing the patient’s life, such as anxiety and psychological stress.

## 4. Discussion

The significant findings that can be extrapolated from the analysis of the papers in this narrative review are geared towards the enhancement of “omics science” (at different levels: genomics, transcriptomics, proteomics, and metabolomics) in the process of discovering biomarkers that are important in determining the development of carotid stenosis [16,17,18,19,20,21,22,23,24,25,26,27,28,29,30,31,32,33,34,35], in defining the vulnerability of the plaque responsible for adverse events [18,19,20,21,22,23,24,25,26,27,28,29,30,31,32,33,34,35,36,37,38,39,40,41,42,43,44,45,46,47,48,49,50], and in describing the outcomes of revascularization interventions [25,26,27,28,29,30,31,32]. In addition, it emerged that the psychosocial sphere is crucial in developing carotid stenosis for a global understanding of the disease, moving from the microscopic world and molecular analysis of pathological mechanisms to the macroscopic world where subjects coexist with the external environment [53,54,55,56]. For ease of reading, the discussion is divided into the following subsections:

### 4.1. The Role of Genomics in the Onset, Progression, and Outcome of Carotid Stenosis

As seen from the studies analyzed, genomics can offer a valuable tool to define the development of carotid stenosis [16,17], determine atherosclerotic plaque instability [18,19,20,21], relate the disease to other comorbidities [22,23,24], and define the outcomes of revascularization interventions [25]. Genomics plays a crucial role in cardiovascular diseases (CVDs) by providing insights into the genetic basis of various cardiovascular conditions, such as cardiomyopathies, coronary artery disease, hypertension, heart failure, stroke, hyperlipidemia, and carotid stenosis [59,60]. Understanding the genetic factors associated with CVD is essential as it allows for the identification of genetic markers linked to variability in the drug response, outcomes, and adverse events related to cardiovascular medications [61]. Moreover, genomics enables the development of personalized or precision cardiovascular approaches tailored to prevent, diagnose, and manage CVDs based on individual genetic profiles [62]. Genomics is also essential in the risk prediction of CVD by developing polygenic risk scores that consider the combined effects of common genetic variants across the genome [63]. Our understanding and treatment approaches for vascular diseases like CS benefit significantly from the knowledge provided by genomics [64]. Genomics plays a crucial role in analyzing the progression and stability of carotid plaque through high-throughput genomic technologies that have enhanced the identification of multiplex genetic polymorphism [63]. Genomics approaches are pivotal in comprehending the development of carotid stenosis, atherosclerotic plaque instability, cardiovascular comorbidities, and the outcomes of revascularization interventions [65]. Several genetic variations are associated with increased susceptibility to carotid stenosis: for example, variants in the APOE gene (apolipoprotein E), particularly the ε4 allele, are linked to higher cholesterol levels and an increased risk of atherosclerosis, including carotid stenosis [66]. In inflammation-related genes, polymorphisms in the interleukin-6 **(**IL6) gene can influence the inflammatory response, contributing to atherosclerotic plaque formation [67]. Genetic variations in the C-reactive protein (CRP) gene can affect plasma levels of CRP, a marker of inflammation and cardiovascular risk [68]. Pathological examinations of the at-risk lesions revealed that plaques containing a high amount of lipids and exhibiting significant inflammation, contributing to mild or moderate narrowing of the arteries, are more likely to rupture, resulting in acute events [69,70]. Detection of these vulnerable plaques assists in categorizing patients at high risk of experiencing acute vascular events. Conventional imaging techniques based on plaque appearance and size are unreliable in predicting the risk of rupture, so the genomic approach has become very important [71,72]. Genome studies also play a role in the assessment of cardiovascular comorbidities linked to carotid stenosis, particularly for diabetes and ischemic stroke, suggesting a more efficient approach to enhancing patient outcomes [73]. Genetic factors can influence the outcomes of carotid interventions such as carotid endarterectomy (CEA) and carotid artery stenting (CAS), so researchers can gain insights into how patients respond to different revascularization procedures and identify potential predictors of treatment success or complications [74]. For example, a study examined the influence of mannose-binding lectin (MBL) on the recurrence of stenosis following eversion endarterectomy in individuals with severe carotid atherosclerosis, suggesting that restenosis after carotid endarterectomy is partly determined by genetics, and indicating that MBL substantially contributes to the pathophysiology of this condition [74,75]

Gaining an understanding of the genetic architecture of cardiovascular diseases requires a comprehensive approach that considers interactions between multiple genes and the environment [76]. Risk factors for cardiovascular diseases, like diabetes, the diet, smoking, stress, a sedentary life, and the circadian rhythm, often lead to changes in epigenetic factors [77]. This indicates that epigenetics regulates genetic and environmental influences [78]. The reversible nature of epigenetic modifications offers the potential to control and revert certain traits, such as plaque vulnerability, suggesting their possible use in CS assessment and treatment [79]. Somatic mutations have been implicated in atherogenesis and vascular disease development, so the role of genomics in atherosclerotic plaque formation is essential [79,80]. Additionally, genetic variations in genes implicated in the mechanisms responsible for vascular disease, such as CS, contribute to different human phenotypes in disease manifestation [81], raising the potential for genomics in precision medicine [76]. 

### 4.2. The Role of Transcriptomics in the Onset, Progression, and Outcome of Carotid Stenosis

Transcriptomics, the study of the complete set of RNA transcripts produced by the genome, has indeed advanced our understanding of the molecular mechanisms underlying carotid stenosis [26,27], atherosclerotic plaque instability [28,29,30,31], and the outcomes of carotid surgery [32]. Research into the simultaneous expression of transcripts related to immunoglobulins, B-lymphocytes, matrix metalloproteinases, and interferon response genes has led to a broader understanding of the processes leading to carotid stenosis and atherosclerotic plaque instability [81,82,83]. The analysis of differentially expressed genes provided further evidence supporting the importance of inflammation, inhibition of cell growth and movement, and increased cellular self-destruction (apoptosis) [83]. Thus, the RNA profile of ruptured plaques contains many transcripts related to inflammation and the reduction in fibrous cap thickness. This reinforces the need for further research into the involvement of B-lymphocytes and interferons in atherosclerotic plaque rupture, and transcriptomics can contribute considerably [84]. mRNA analysis of genes involved in inflammation, such as NLRP3 (NOD-like receptor protein 3), interleukin-1 beta (IL-1β), and tumor necrosis factor-alpha (TNF-α), revealed they are significantly upregulated in unstable plaques. These genes contribute to the inflammatory milieu destabilizing carotid plaques [85].

The sophisticated and detailed analysis of genetic changes at the transcriptome level in damaged blood vessels from animal disease models and human samples lays the groundwork for identifying new potential treatment targets for restenosis [86]. Additionally, these analyses help to discover reliable biomarkers in the bloodstream that could predict restenosis following medical interventions in patients, potentially facilitating early diagnosis and treatment [87]. MicroRNAs (miRNAs) are small, non-coding RNA molecules that regulate gene expression post-transcriptionally. They play a crucial role in modulating the molecular pathways involved in carotid stenosis: regulation of inflammation, plaque stability, remodeling, and diagnostic and prognostic biomarkers [88].

MicroRNAs, long non-coding RNAs, and circular RNAs are emphasized in regulating gene expression and their potential function in atherosclerotic plaque formation [89,90]. MicroRNAs have been identified as crucial regulators in vascular diseases, influencing disease progression and potential therapeutic targets [91]. Studies have shown that long non-coding RNAs are regulatory in vascular dysfunction and atherosclerosis, highlighting their potential as therapeutic targets for treating vascular diseases [92]. Translating basic transcriptomic research into clinical applications has recently become a new challenge, and further studies are being requested [93]. 

### 4.3. The Role of Proteomics in the Onset, Progression, and Outcome of Carotid Stenosis

By analyzing the protein composition of tissues and bodily fluids, proteomics offers valuable insights into the molecular mechanisms underlying CS [32,37,44]. Proteomic studies have revealed changes in the vascular proteomes of men and women [94]. Gender plays a role in cardiovascular risk. Women have better HDL levels and lower ferritin levels than men but develop cardiovascular disease around ten years later [95]. After menopause, women’s cardiovascular disease progresses rapidly. Women with carotid stenosis have more stable plaques than men [95,96]. Men’s plaques have higher cellularity, inflammation, and neovascularization [97]. Proteomics in vascular disease research has identified novel proteins associated with developing and progressing atherosclerotic plaques, a common cause of CS [98]. The proteome can provide crucial information about the pathophysiology of vascular diseases, aiding in discovering new therapeutic targets and biomarkers [99]. Proteomic approaches have been instrumental in investigating the molecular changes that drive the activation of vascular smooth muscle cells, shedding light on the transition from a quiescent to a proliferative phenotype or from stable plaque to unstable plaque [100]. Moreover, proteomic research has been pivotal in elucidating oxidative stress and inflammation in conditions like CS [101]. Accordingly, by measuring specific protein markers associated with oxidative stress and inflammation, researchers can gain insights into the pathogenesis of vascular diseases and identify potential targets for precision medicine (PM) [100,101]. Various types of MMPs, analyzed by proteomics, have been associated with atherosclerotic plaques in CS. Particularly, through a proteomic approach, the interaction between MMP-9 and NGAL (neutrophil gelatinase-associated lipocalin) has been associated with plaque vulnerability, and MMP-9/NGAL has been proposed as a target of statins in patients with CS [102]. The relationship between MMP-9 and NGAL is also studied in the context of arterial aneurysm rupture and patients with pulmonary embolism [103,104,105]. More research is needed to understand better the role of MMPs in the cardiovascular system, particularly in post-revascularization procedures such as CEA and CAS, and proteomic approaches may provide valuable insights [105,106].

### 4.4. The Role of Metabolomics in the Onset, Progression, and Outcome of Carotid Stenosis

Metabolomics is essential in cardiovascular research for its insights into disease mechanisms, identifying biomarkers, and improving diagnostic and therapeutic strategies [45,46,47,48,49,50,51]. Particularly, metabolomics technologies in CS have enabled a deeper investigation into the metabolic pathways involved in disease, providing insights into how metabolite alterations contribute to atherosclerotic plaque characteristics [107,108]. Metabolomics plays a significant role in determining the stability of carotid plaques by offering insights into the metabolic processes and pathways that determine plaque formation, progression, and rupture [108]. Recently, a plaque-specific genome-scale metabolic network (GEM) was proposed with a high ability to accurately forecast irregularities in cholesterol hydroxylation, inositol metabolism, and the glutamine/glutamate pathway in rupture-prone hemorrhaged carotid plaques, potentially leading to new diagnostic or therapeutic approaches for plaque stabilization [109]. By studying metabolomic profiles, researchers can uncover metabolites associated with various cardiovascular conditions, aiding in the early detection and management of cardiovascular diseases [110]. Metabolomics has been crucial in discovering metabolic biomarkers for clinical diagnosis, early warning, and targeting molecular pathogenesis [111]. Targeted metabolomics methods have identified new cardiovascular disease risk molecular markers, linking exposures such as dietary intake and the microbiota with cardiometabolic traits [112]. Integrating metabolomics and gut microbiota studies offers valuable insights into the carotid stenosis pathophysiology. Metabolic signatures, microbial compositions, and genetic factors associated with the disease can advance knowledge of carotid artery atherosclerosis and guide personalized treatment approaches [107,108]. Cardio-metabolomics is a new direction in cardiovascular science that allows researchers to study changes in the metabolome and metabolic networks in cardiovascular diseases [112]. Cardio-metabolomic studies have identified associations between metabolites and CS risk factors, offering valuable information on the development of atherosclerosis in CS [113].

### 4.5. The Role of Social Aspects in Carotid Stenosis

The results of this review highlight that the social aspect plays a crucial role in cardiovascular diseases such as CS [55,56,57,58]; for this reason, various social determinants of health (SDHs) could be considered new disease markers [114]. Social networks and engagement have been associated with cardiovascular disease risk, influencing disease onset, patient survival, and mortality [115,116]. Understanding cardiovascular disease’s social and behavioral aspects is essential to prevent risk factors such as a lipid diet, lack of physical activity, smoking, and stress and to manage these conditions effectively [117]. Social determinants of health (SDHs), including racial/cultural identity, gender identity, community, and the social context, have a significant influence on cardiovascular morbidity and mortality [118]. Social support, social ties, and marital status have been linked to cardiovascular risk, health behaviors, and disease outcomes [119,120]. Social isolation or destitution can increase the CS incidence [121]. Psychosocial risk factors, including chronic psychological and social stress, can impact the development and course of cardiovascular disease by influencing physiological processes such as serum cholesterol levels, platelet aggregation, and heart rate variability [122]. The association between education and cardiovascular disease incidence is mediated by factors such as hypertension, diabetes, and body mass index, highlighting the need to address social determinants to reduce the possibility of a negative cardiovascular disease diagnosis [123].

### 4.6. Circulating Biomarkers and Omics Biomarkers in Current Clinical Practice

In current clinical practice, several circulating biomarkers provide less invasive, more accessible, and inexpensive tools for assessing the disease status and monitoring the treatment response in patients with carotid stenosis [124]. Biomarkers like tumor necrosis factor-stimulated gene-6 (TSG-6) and lipoprotein-associated phospholipase A2 (Lp-PLA2) have shown promise in non-invasively screening for severe carotid stenosis and assessing plaque vulnerability, respectively [124,125]. The levels of serum TSG-6 were found to be more effective in diagnosing severe and symptomatic carotid stenosis compared to other biomarkers (*p* < 0.05), particularly in the identification of symptomatic stenosis (*p* < 0.01) [124]. The plasma concentrations of Lp-PLA2 show a positive correlation (*p* < 0.05) with intraplaque angiogenesis, and its levels can indicate the stability of carotid plaques [125].

Additionally, a notable link between carotid stenosis and high-sensitivity C-reactive protein (hs-CRP), soluble vascular cell adhesion molecule 1 (sVCAM-1), and interleukin-6 (IL-6 levels) was revealed, indicating that these inflammatory biomarkers could be associated with atherosclerosis, and their levels in subjects who underwent to carotid endarterectomy were elevated (*p* < 0.001) [126].

N-terminal pro-B-type natriuretic peptide and midregional pro-adrenomedullin are predictors for clinically detected carotid artery stenosis during long-term follow-up [127]. The platelet-to-lymphocyte ratio (PLR) has been proposed to assess the severity of stenosis and stroke in patients with carotid arterial disease [128]. High preoperative neutrophil-to-lymphocyte ratios (NLRs) are significantly associated with symptomatic carotid stenosis in patients who have undergone carotid endarterectomy. In particular, symptomatic patients (64.2%, *p* = 0.005) had a higher percentage in the group with NLR > 2.95, suggesting a positive association between pre-surgery NLR and symptomatic CEA patients [129]. Elevated levels of inflammatory and oxidative stress biomarkers such as monocyte chemotactic protein-4 (MCP-4), amyloid A (AA), and tumor necrosis factor-alpha (TNF-α) (*p* < 0.001) have been associated with severe carotid artery stenosis and multivessel coronary arteries in elderly individuals. Monitoring oxidative stress and inflammation biomarkers could offer a promising approach to developing an effective method for assessing the severity of carotid artery and coronary artery stenosis [130]. Moreover, increased endothelial activation has been observed in patients with recently symptomatic carotid artery stenosis, indicating the potential value of endothelial biomarkers in predicting stroke risk in carotid stenosis patients [131]. 

In managing carotid stenosis, the comparison between omics and circulating biomarkers plays a significant role in enhancing risk assessment, diagnosis, and treatment strategies. Omics biomarkers derived from high-throughput technologies like genomics, transcriptomics, proteomics, and metabolomics offer a comprehensive understanding of the molecular mechanisms underlying carotid stenosis [132]. These biomarkers can aid in identifying vulnerable plaques and predicting the risk of ischemic events, thereby guiding clinical decision-making for interventions such as carotid revascularization [133]. Integrating omics data with clinical parameters can lead to developing predictive models for stratifying patients with carotid stenosis based on their risk profiles [134]. The combination of circulating and omics biomarkers offers a comprehensive approach to characterizing carotid stenosis, from molecular pathways to clinical outcomes [133,134]. 

Integrating omics and circulating biomarkers in managing carotid stenosis holds promise for improving risk prediction, early detection of vulnerable plaques, and personalized treatment strategies. By leveraging the strengths of both types of biomarkers, clinicians can enhance patient care and outcomes in individuals with carotid stenosis [134].

### 4.7. “Omics Science” and the Paradigm of Complexity in the Research on New Biomarkers

By combining genomics, proteomics, and metabolomics, researchers have gained a valuable understanding of the causes and mechanisms of vascular diseases. This has revealed connections between variability in omics profiles and clinical traits, suggesting a translation of these findings into practical applications [135]. Studies on various molecular levels, such as transcriptomic, proteomic, metabolomic, and lipidomic research, have improved our knowledge of atherosclerotic plaques, their diversity, and their adaptability. This has led to the development of new hypotheses for mechanistic studies and potential new biomarker discovery [135,136]. It is important to note that in addition to biomarkers that can be derived from “omics science”, there are also instrumental markers of carotid stenosis, including transcranial Doppler detection of stenosis-induced micro-embolism in the cerebral vasculature, assessment of collateral cerebral blood flow [137], and evaluation of the degree of cerebral hypoperfusion, which could contribute to assessing the severity, progression, and outcomes of carotid stenosis [138]. By identifying and utilizing these biomarkers associated with those derived from omics science, healthcare providers can enhance risk stratification, personalize treatment approaches, and improve patient care in individuals with carotid artery disease [135,136,137,138].

**Figure 1:**
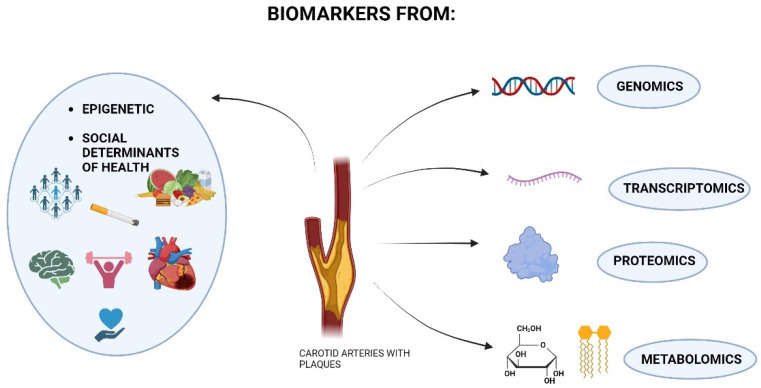
Schematic representation of the research areas from which biomarkers of carotid stenosis can be obtained. Biomarkers can come from ‘‘omics‘‘ science, genomics, transcriptomics (RNA level), proteomics, and metabolomics. In addition, markers from epigenetics may also be valuable, considering the environmental context of the patient affected by carotid stenosis and disease-related risk factors (diet, low physical activity, smoking, and other comorbidities such as heart disease). Social determinants of health can be counted as new biomarkers, including all aspects related to the psychosocial well-being of the individual that have consequences for the onset of carotid stenosis: interactions with others, psychological stress, socio-cultural context, education, and financial income.

In the microscopic world, proteomics studies have yielded various cellular proteins. Despite our current knowledge, the interactions and spatial organization of proteins within the cell’s functional modules, often referred to as the cell’s “molecular sociology”, are still not understood, and further studies are requested to fully comprehend the complexity of the communication between cells’ proteins and the external environment [139]. The same concept is transferred to the macroscopic world, characterized by complex interactions between human beings and external stimuli. From Morin’s theory about ‘‘the paradigm of complexity”, complexity recognizes the interconnectedness of individuals with the natural world and rejects conceptually isolating humans from nature [140]. This perspective allows for new connections between biology, anthropology, and sociology because it recognizes fundamental relationships between seemingly unrelated aspects of human life [141]. Living systems are complex, disordered, and self-organizing, responding to various combinations of environmental factors not governed by genetic material [140]. Open systems foster biological and cultural evolution interactions, interweaving both processes [142]. From these perspectives, education, socioeconomic status (SES), and other social themes play a significant role in cardiovascular risk, disease incidence, and health outcomes, opening up the possibility of introducing them into mathematical systems that can calculate not only the patient’s physical state but also their psychosocial one [114]. Integrating epigenetics, related to environmental factors and social determinants of health, with “omics sciences” is an exciting approach for biomarker discovery in cardiovascular diseases like CS [143,144], as shown in Figure 1.

The complexity theory surpasses the limitations of traditional knowledge, positivism, Newtonian physics, and symmetric mathematics. Its foundation lies in recognizing the social determinants of health and the significance of the organizational culture [140]. Health systems are considered complex adaptive systems [142]. Furthermore, social determinants of health could assist in predicting disease outcomes in CS, offering valuable insights for healthcare professionals in diagnosing and how patients may respond to treatment [114,115,116,117,118,119,120,121,122,123,124,125,126,127,128,129,130,131,132,133,134,135,136,137,138,139,140,141,142,143]. This interdisciplinary and transversal method of interaction between biological and social sciences, initially proposed by Edgar Morin [140], should be further explored and implemented in the clinical setting to improve the well-being and management of patients through ‘‘omics‘‘ and social biomarkers.

## 5. Conclusions

This narrative review has provided a detailed analysis of genomic, transcriptomic, proteomic, and metabolomic technologies in revealing new biomarkers for carotid stenosis. Various genes, transcriptomes (at the RNA level), proteins (i.e., metalloproteinases), and metabolites have been identified as potential biomarkers for diagnosing, preventing, and treating carotid stenosis. This review has also explored the correlation of these biomarkers with the atherosclerotic plaque composition, its morphological characteristics, and the outcomes following carotid revascularization procedures. The connection between carotid stenosis and concurrent conditions like diabetes mellitus, as well as the potential link with microbiota and disparities based on gender, have been considered. The social dimension of carotid stenosis has also been addressed from a multidisciplinary and cross-sectional perspective, considering social determinants of cardiovascular health integrated in the context of emerging biomarkers from “omics” sciences. The importance of biomarkers revealed by genetic, RNA, protein, and metabolomic approaches has been emphasized; particular relevance has been given to social biomarkers embedded in a broader cultural context, theorized by Morin’s paradigm of complexity. The great potential of the detection techniques used in omics sciences has also been discussed. However, further research is needed to translate these findings into modern clinical practice, enabling more efficient patient management in precision medicine.

## Figures and Tables

**Table 1:** SANRA (Scale for the Assessment of Narrative Review Articles) quality assessment related to genomics and carotid stenosis articles selected.

ITEMS	Justification of the Article’s Importance for the Readership	Statement of Concrete Aims or Formulation of Questions	Description of the Literature Search	Referencing	Scientific Reasoning	Appropriate Presentation of Data	Total Score
	- The importance is not justified (score 0) - The importance is alluded to but not explicitly justified (score 1) - The importance is explicitly justified (score 2)	- No aims or questions are formulated (score 0) - Aims are formulated generally but not concretely or in terms of clear questions (score 1) - One or more concrete aims or questions are formulated (score 2)	- The search strategy is not presented (score 0) - The literature search is described briefly (score 1) - The literature search is described in detail (score 2)	- References do not support key statements (score 0) - The referencing of key statements is inconsistent (score 1) - References support key statements (score 2)	- The article’s point is not based on appropriate arguments (score 0) - Appropriate evidence is introduced selectively (score 1) - Appropriate evidence is generally present (score 2)	- Data are presented inadequately (score 0) - Data are often not presented in the most appropriate way (score 1) - Relevant outcome data are presented appropriately (score 2)	
Yi et al. [16]	Score: 2	Score: 2	Score: 2	Score: 2	Score: 2	Score: 2	12
Wang et al. [17]	Score: 1	Score: 2	Score: 1	Score: 2	Score: 2	Score: 2	10
Li et al. [18]	Score: 2	Score: 2	Score: 2	Score: 2	Score: 2	Score: 2	12
Straface et al. [19]	Score: 2	Score: 2	Score: 2	Score: 2	Score: 2	Score: 2	12
Salem et al. [20]	Score: 1	Score: 2	Score: 1	Score: 2	Score: 2	Score: 2	10
Vasuri et al. [21]	Score: 1	Score: 2	Score: 1	Score: 2	Score: 2	Score: 2	10
Kostulas et al. [22]	Score: 2	Score: 2	Score: 2	Score: 2	Score: 2	Score: 2	12
Yi et al. [23]	Score: 1	Score: 2	Score: 1	Score: 2	Score: 2	Score: 2	10
Wu et al. [24]	Score: 2	Score: 2	Score: 2	Score: 2	Score: 1	Score: 1	10
Yocum et al. [25]	Score: 2	Score: 2	Score: 2	Score: 2	Score: 2	Score: 2	12

**Table 2:** SANRA (Scale for the Assessment of Narrative Review Articles) quality assessment related to transcriptomics and carotid stenosis articles selected.

ITEMS	Justification of the Article’s Importance for the Readership	Statement of Concrete Aims or Formulation of Questions	Description of the Literature Search	Referencing	Scientific Reasoning	Appropriate Presentation of Data	Total Score
	- The importance is not justified (score 0) - The importance is alluded to but not explicitly justified (score 1) - The importance is explicitly justified (score 2)	- No aims or questions are formulated (score 0) - Aims are formulated generally but not concretely or in terms of clear questions (score 1) - One or more concrete aims or questions are formulated (score 2)	- The search strategy is not presented (score 0) - The literature search is described briefly (score 1) - The literature search is described in detail (score 2)	- References do not support key statements (score 0) - The referencing of key statements is inconsistent (score 1) - References support key statements (score 2)	- The article’s point is not based on appropriate arguments (score 0) - Appropriate evidence is introduced selectively (score 1) - Appropriate evidence is generally present (score 2)	- Data are presented inadequately (score 0) - Data are often not presented in the most appropriate way (score 1) - Relevant outcome data are presented appropriately (score 2)	
Kim et al. [26]	Score: 2	Score: 2	Score: 2	Score: 2	Score: 2	Score: 2	12
Perisic et al. [27]	Score: 2	Score: 2	Score: 2	Score: 1	Score: 1	Score: 2	10
Luo et al. [28]	Score: 2	Score: 2	Score: 2	Score: 2	Score: 2	Score: 2	12
Karlof et al. [29]	Score: 2	Score: 2	Score: 2	Score: 2	Score: 2	Score: 2	12
Tan et al. [30]	Score: 1	Score: 2	Score: 1	Score: 2	Score: 2	Score: 2	10
Perisic et al. [31]	Score: 1	Score: 1	Score: 2	Score: 1	Score: 2	Score: 2	10
Forte et al. [32]	Score: 2	Score: 2	Score: 2	Score: 2	Score: 2	Score: 2	12
Deng et al. [33]	Score: 2	Score: 2	Score: 2	Score: 2	Score: 2	Score: 2	12
Chen et al. [34]	Score: 2	Score: 2	Score: 1	Score: 1	Score: 2	Score: 2	10

**Table 3:** SANRA (Scale for the Assessment of Narrative Review Articles) quality assessment related to proteomics and carotid stenosis articles selected.

ITEMS	Justification of the Article’s Importance for the Readership	Statement of Concrete Aims or Formulation of Questions	Description of the Literature Search	Referencing	Scientific Reasoning	Appropriate Presentation of Data	Total Score
	- The importance is not justified (score 0) - The importance is alluded to but not explicitly justified (score 1) - The importance is explicitly justified (score 2)	- No aims or questions are formulated (score 0) - Aims are formulated generally but not concretely or in terms of clear questions (score 1) - One or more concrete aims or questions are formulated (score 2)	- The search strategy is not presented (score 0) - The literature search is described briefly (score 1) - The literature search is described in detail (score 2)	- References do not support key statements (score 0) - The referencing of key statements is inconsistent (score 1) - References support key statements (score 2)	- The article’s point is not based on appropriate arguments (score 0) - Appropriate evidence is introduced selectively (score 1) - Appropriate evidence is generally present (score 2)	- Data are presented inadequately (score 0) - Data are often not presented in the most appropriate way (score 1) - Relevant outcome data are presented appropriately (score 2)	
Forte et al. [32]	Score: 2	Score: 2	Score: 2	Score: 2	Score: 2	Score: 2	12
Theofilatos et al. [35]	Score: 2	Score: 2	Score: 2	Score: 2	Score: 2	Score: 2	12
Liang et al. [36]	Score: 1	Score: 1	Score: 2	Score: 2	Score: 2	Score: 2	10
Porcelli et al. [37]	Score: 1	Score: 2	Score: 1	Score: 2	Score: 2	Score: 2	10
Wang et al. [38]	Score: 1	Score: 1	Score: 2	Score: 2	Score: 2	Score: 2	10
Langley et al. [39]	Score: 2	Score: 2	Score: 2	Score: 2	Score: 2	Score: 2	12
Hao et al. [40]	Score: 1	Score: 1	Score: 2	Score: 2	Score: 2	Score: 2	10
Baragetti et al. [41]	Score: 1	Score: 1	Score: 2	Score: 2	Score: 2	Score: 2	10
Lorentsen et al. [42]	Score: 2	Score: 2	Score: 2	Score: 2	Score: 2	Score: 2	12
Lai et al. [43]	Score: 2	Score: 2	Score: 2	Score: 2	Score: 2	Score: 2	12
Lepedda et al. [44]	Score: 2	Score: 1	Score: 1	Score: 2	Score: 2	Score: 2	10

**Table 4:** SANRA (Scale for the Assessment of Narrative Review Articles) quality assessment related to metabolomics and carotid stenosis articles selected.

AUTHORS	Justification of the Article’s Importance for the Readership	Statement of Concrete Aims or Formulation of Questions	Description of the Literature Search	Referencing	Scientific Reasoning	Appropriate Presentation of Data	Total Score
	- The importance is not justified (score 0) - The importance is alluded to but not explicitly justified (score 1) - The importance is explicitly justified (score 2)	- No aims or questions are formulated (score 0) - Aims are formulated generally but not concretely or in terms of clear questions (score 1) - One or more concrete aims or questions are formulated (score 2)	- The search strategy is not presented (score 0) - The literature search is described briefly (score 1) - The literature search is described in detail (score 2)	- References do not support key statements (score 0) - The referencing of key statements is inconsistent (score 1) - References support key statements (score 2)	- The article’s point is not based on appropriate arguments (score 0) - Appropriate evidence is introduced selectively (score 1) - Appropriate evidence is generally present (score 2)	- Data are presented inadequately (score 0) - Data are often not presented in the most appropriate way (score 1) - Relevant outcome data are presented appropriately (score 2)	
Lee et al. [45]	Score: 2	Score: 2	Score: 2	Score: 2	Score: 2	Score: 2	12
Azzini et al. [46]	Score: 2	Score: 2	Score: 2	Score: 2	Score: 2	Score: 2	12
Lin et al. [47]	Score: 1	Score: 1	Score: 2	Score: 2	Score: 2	Score: 2	10
Liu et al. [48]	Score: 2	Score: 1	Score: 1	Score: 2	Score: 2	Score: 2	10
Mas et al. [49]	Score: 2	Score: 1	Score: 2	Score: 1	Score: 2	Score: 2	10
Vorkas et al. [50]	Score: 2	Score: 2	Score: 2	Score: 2	Score: 2	Score: 2	12
Vorkas et al. [51]	Score: 1	Score: 1	Score: 2	Score: 2	Score: 2	Score: 2	10
Stegemann et al. [52]	Score: 1	Score: 2	Score: 2	Score: 1	Score: 2	Score: 2	10
Wang et al. [53]	Score: 2	Score: 2	Score: 2	Score: 2	Score: 2	Score: 2	12
Cason et al. [54]	Score: 1	Score: 1	Score: 2	Score: 2	Score: 2	Score: 2	10

**Table 5:** SANRA (Scale for the Assessment of Narrative Review Articles) quality assessment related to social aspects and carotid stenosis articles selected.

AUTHORS	Justification of the Article’s Importance for the Readership	Statement of Concrete Aims of Formulation of Questions	Description of the Literature Search	Referencing	Scientific Reasoning	Appropriate Presentation of Data	Total Score
	- The importance is not justified (score 0) - The importance is alluded to but not explicitly justified (score 1) - The importance is explicitly justified (score 2)	- No aims or questions are formulated (score 0) - Aims are formulated generally but not concretely or in terms of clear questions (score 1) - One or more concrete aims or questions are formulated (score 2)	- The search strategy is not presented (score 0) - The literature search is described briefly (score 1) - The literature search is described in detail (score 2)	- References do not support key statements (score 0) - The referencing of key statements is inconsistent (score 1) - References support key statements (score 2)	- The article’s point is not based on appropriate arguments (score 0) - Appropriate evidence is introduced selectively (score 1) - Appropriate evidence is generally present (score 2)	- Data are presented inadequately (score 0) - Data are often not presented in the most appropriate way (score 1) - Relevant outcome data are presented appropriately (score 2)	
Hsu et al. [55]	Score: 2	Score: 2	Score: 2	Score: 2	Score: 2	Score: 2	12
Wu et al. [56]	Score: 2	Score: 2	Score: 2	Score: 2	Score: 2	Score: 2	12
Baxi et al. [57]	Score: 2	Score: 2	Score: 2	Score: 2	Score: 2	Score: 2	12
Aber et al. [58]	Score: 2	Score: 1	Score: 1	Score: 2	Score: 2	Score: 2	10

**Table 6:** Principal features of the reviewed articles related to genomics and carotid stenosis.

AUTHORS	TITLE	YEAR OF PUBLICATION	RESEARCH DESIGN	SAMPLE CHARACTERISTICS	MAIN FINDING
Yi et al. [16]	CYP genetic variants, CYP metabolite levels, and symptomatic carotid stenosis in ischemic stroke patients	2016	Experimental study	136 patients with CS ^1^ and ischemic stroke and 158 without CS	Analysis of the differences between the cytochrome P 450 genetic polymorphism and cytochrome P metabolites in patients with symptomatic CS
Wang et al. [17]	Investigation of the underlying genes and mechanism of macrophage-enriched ruptured atherosclerotic plaques using bioinformatics method	2019	Experimental prospective study	6 stable atherosclerotic samples and 5 ruptured samples from the GEO database	Several differentially expressed genes in ruptured and macrophagic atherosclerotic plaques
Li et al. [18]	Comprehensive analysis identifies crucial genes associated with immune cells mediating progression of carotid atherosclerotic plaque	2024	Experimental study	Gene datasets with 16 advanced and 13 early atherosclerotic plaques, 32 atheromas, and 32 intact tissues	The gene PLEK ^5^ was associated with monocyte and macrophage functions in atherosclerotic plaque
Straface et al. [19]	Assessment of the genetic effects of polymorphisms in the osteoprotegerin gene, TNFRSF11B, on serum osteoprotegerin levels and carotid plaque vulnerability	2011	Experimental study	Carotid plaques from 177 patients who underwent CEA and 303 healthy controls	The gene polymorphism of TNFRSF11B ^6^ is associated with unstable carotid plaque
Salem et al. [20]	Gene and protein expression of chemokine (C-C-Motif) ligand 19 is upregulated in unstable carotid atherosclerotic plaques	2016	Experimental prospective study	24 sample plaques from patients who underwent CEA ^2^	Upregulation of the CCL19 ^3^ gene in unstable atherosclerotic plaques
Vasuri et al. [21]	Gene polymorphism in tissue epidermal growth factor receptor (EGFR) influences clinical and histological vulnerability of carotid plaques	2022	Retrospective study	29 patients who underwent CEA	The polymorphism observed for the EGFR ^4^ gene is associated with protection against plaque hemorrhage and could, therefore, be a determinant of pre-surgical decision-making
Kostulas et al. [22]	Genetic profile of ischemic cerebrovascular disease and carotid stenosis	2008	Experimental prospective study	928 patients with ischemic stroke and CS, and 602 healthy patients	Gene polymorphisms related to the factor VII gene, apolipoprotein E gene, and two renin gene polymorphisms were associated with ischemic stroke and CS
Yi et al. [23]	The txa2r rs1131882, p2y1 rs1371097 and gpiiia rs2317676 three-loci interactions may increase the risk of carotid stenosis in patients with ischemic stroke	2019	Experimental prospective study	236 ischemic stroke patients with CS ^1^ and 378 ischemic stroke patients without CS	Gene interaction among three different loci related to platelet activation and responsible for the higher risk of CS in ischemic stroke patients
Wu et al. [24]	Associations of genetic markers of diabetes mellitus with carotid atherosclerosis: A community-based case–control study	2023	Case–control study	309 patients with carotid plaque and 439 healthy controls	The positive association between nine single-nucleotide polymorphisms related to diabetes mellitus and CS
Yocum et al. [25]	Inducible nitric oxide synthase promoter polymorphism affords protection against cognitive dysfunction after carotid endarterectomy	2009	Experimental prospective study	185 patients who underwent CEA and 60 in the control group	A polymorphism within the promoter region of iNOS ^7^ protects from moderate to severe cognitive dysfunction 1 month after CEA

^1^ Carotid stenosis; ^2^ carotid endarterectomy; ^3^ chemokine (c-c motif) ligand 19 (CCL19); ^4^ epidermal growth factor receptor; ^5^ pleckstrin; ^6^ osteoprotegerin gene; ^7^ inducible nitric oxide synthase.

**Table 7:** Principal features of the reviewed articles related to transcriptomics and carotid stenosis.

AUTHORS	TITLE	YEAR OF PUBLICATION	RESEARCH DESIGN	SAMPLE CHARACTERISTICS	MAIN FINDING
Kim et al. [26]	Macrophage–hypoxia-inducible factor-1α signaling in carotid artery stenosis. *American Journal of Pathology*	2021	Experimental study	Macrophage cell line culture for in vitro studies and mice with ligated carotid for in vivo studies	Controlling the interactions between macrophages and HIF1α ^1^ could halt the advancement of CS ^2^
Perisic et al. [27]	Profiling of atherosclerotic lesions by gene and tissue microarrays reveals PCSK6 as a novel protease in unstable carotid atherosclerosis	2013	Experimental study	127 plaques selected from asymptomatic and symptomatic patients who underwent carotid surgery	Transcriptomic method to identify proteins, like PCFK6 ^3^, differentially expressed in plaques from symptomatic patients
Luo et al. [28]	Eif2α mediated integrated stress response connects multiple intracellular signaling to reprogram vascular smooth muscle cell fate in carotid plaques	2024	Experimental study	Primary carotid vascular smooth muscle cells derived from carotid atherosclerotic plaques	Morphological and functional modifications in carotid vascular smooth muscle cells could be considerable biomarkers for CS
Karlöf et al. [29]	Correlation of computed tomography with carotid plaque transcriptomes associates calcification with lesion-stabilization	2019	Experimental study	Plaques calcified from patients who underwent CEA ^4^	Transcriptional evaluation of plaque calcification levels. Calcification is indicative of plaque stability
Tan et al. [30]	Transcriptomics reveals crucial cell subsets and functional heterogeneity associated with carotid atherosclerosis and cerebrovascular events	2023	Experimental study	Atherosclerotic plaque tissue from 20 individuals who underwent CEA	The different types of cells present in atherosclerotic environments may be usable for developing targeted cardiovascular immunotherapies
Perisic et al. [31]	Gene expression signatures, pathways and networks in carotid atherosclerosis	2016	Experimental study	127 plaques and 96 blood samples from asymptomatic and symptomatic patients	Transcriptomic analysis reveals the role of inflammation and proteases in plaque instability, enhancing the risk of stroke
Forte et al. [32]	Expression profiles in surgically-induced carotid stenosis: A combined transcriptomic and proteomic investigation	2008	Experimental study	Rats with carotid arteriotomy	Transcriptional approach to revealing molecular pathways that could be activated after carotid surgery to prevent restenosis
Deng et al. [33]	Differential expression profile of miRNAs between stable and vulnerable plaques of carotid artery stenosis patients	2023	Experimental study	3 patients with stable plaque and 3 patients with unstable plaque	Determination of different transcription profiles, between stable and unstable atherosclerotic plaques
Chen et al. [34]	MicroRNA-214 modulates the senescence of vascular smooth muscle cells in carotid artery stenosis	2020	Experimental study	Blood samples from patients with CS and aortic vascular smooth muscle cells from rats for in vitro studies	miR-214 (microRNA 214) induces senescence of vascular smooth muscle cells and cell death, resulting in CS

^1^ Myeloid-hypoxia-inducible factor-1α; ^2^ carotid stenosis; ^3^ proprotein convertase subtilisin/kexin type 6; ^4^ carotid endarterectomy.

**Table 8:** Principal features of the reviewed articles related to proteomics and carotid stenosis.

AUTHORS	TITLE		RESEARCH DESIGN	SAMPLE CHARACTERISTICS	MAIN FINDING
Forte et al. [32]	Expression profiles in surgically-induced carotid stenosis: A combined transcriptomic and proteomic investigation	2008	Experimental study	Rats with carotid arteriotomy	Differential protein pattern in carotid artery contractility after CEA ^1^ intervention that may lead to restenosis
Theofilatos et al. [35]	Proteomic atlas of atherosclerosis: The contribution of proteoglycans to sex differences, plaque phenotypes, and outcomes	2023	Experimental study	Atherosclerotic plaques from 120 patients who underwent CEA	Proteomic signatures to understand how proteoglycans contribute to the differences between sexes, plaque characteristics, and outcomes in CS ^2^
Liang et al. [36]	Distinctive proteomic profiles among different regions of human carotid plaques in men and women	2016	Experimental study	Atherosclerotic plaques from 26 patients who underwent CEA	Proteomic differences between male and female patients with CS
Porcelli et al. [37]	Proteomic analysis of atherosclerotic plaque	2010	Experimental study	Atherosclerotic plaques from 10 patients who underwent CEA	Analysis of protein profile responsible for genesis and progression of atherosclerotic plaque in CS
Wang et al. [38]	Urinary proteomics identifying novel biomarkers for the diagnosis and phenotyping of carotid artery stenosis	2021	Experimental study	Urine samples from patients with CS who underwent CEA	Different urinary proteins could differentiate between symptomatic and asymptomatic patients
Langley et al. [39]	Extracellular matrix proteomics identifies molecular signature of symptomatic carotid plaques	2017	Experimental study	Atherosclerotic plaques from 12 patients who underwent CEA: 6 asymptomatic and 6 symptomatic patients	Proteomic analysis revealed the presence of high levels of MMP-9 ^3^ in atherosclerotic plaques of symptomatic patients with CS
Hao et al. [40]	Deep proteomic profiling of human carotid atherosclerotic plaques using multidimensional LC-MS/MS	2014	Experimental study	Atherosclerotic plaques from 38 patients who underwent CEA	Using LC-MS/MS ^4^, it was possible to assess the presence of many atherosclerotic plaque-derived proteins, including metalloproteinases and their inhibitors
Baragetti et al. [41]	Targeted plasma proteomics to predict the development of carotid plaques	2022	Longitudinal observational analysis	Plasma from 586 subjects with carotid atherosclerosis	Variation in the levels of proteins, including a reduction in MMP-10 ^5^, may predict the appearance of subclinical CS
Lorentzen et al. [42]	Proteomic analysis of the extracellular matrix of human atherosclerotic plaques shows marked changes between plaque types	2024	Experimental study	Plaque sample from 21 patients who underwent CEA	Unstable plaque is rich in proteins associated with inflammation, extracellular matrix (ECM) remodeling, and protein degradation; stable plaque is rich in structural proteins
Lai et al. [43]	Characterization of the proteome of stable and unstable carotid atherosclerotic plaques using data-independent acquisition mass spectrometry	2024	Experimental cohort study	182 patients with CS	Different protein identifies stable and unstable plaques
Lepedda et al. [44]	A proteomic approach to differentiate histologically classified stable and unstable plaques from human carotid arteries	2009	Experimental study	19 stable plaques and 29 unstable plaques from patients with CS who underwent CEA	Proteomic analysis to differentiate stable plaques from unstable plaques populated by proteins with pro-oxidant and pro-inflammatory activity

^1^ Carotid endoarterectomy; ^2^ carotid stenosis; ^3^ metalloproteinase-9; ^4^ liquid chromatography tandem mass spectrometry; ^5^ metalloproteinase-10.

**Table 9:** Principal features of the reviewed articles related to metabolomics and carotid stenosis.

AUTHORS	TITLE	YEAR OF PUBLICATION	RESEARCH DESIGN	SAMPLE CHARACTERISTICS	MAIN FINDING
Lee et al. [45]	Metabolomics study in severe extracranial carotid artery stenosis	2019	Experimental research	130 patients with CS ^1^	Some metabolites related to diet are associated with the pathological mechanism of CS
Azzini et al. [46]	Homocysteine: Its possible emerging role in at-risk population groups	2020	Review	None	Highlights homocysteine, a metabolic marker for CS in male patients
Lin et al. [47]	Identification of metabolomics biomarkers in extracranial carotid artery stenosis	2022	Experimental study	176 male healthy controls and 173 patients with stroke and CS	Through metabolomic analysis, it is possible to distinguish patients with CS from healthy controls
Liu et al. [48]	Association between lipid profiles and presence of carotid plaque	2019	Experimental study	3214 patients with CS	Different lipid profiles are connected to CS, in particular, with the morphology/composition of the plaque
Mas et al. [49]	Local non-esterified fatty acids correlate with inflammation in atheroma plaques of patients with type 2 diabetes	2010	Experimental study	Carotid plaques from 40 patients who underwent CEA ^2^	It is proposed that ^3^ NEFAs are produced at the carotid site in diabetic subjects with CS, suggesting a link between CS and diabetes through this metabolite
Vorkas et al. [50]	Metabolic phenotyping of atherosclerotic plaques reveals latent associations between free cholesterol and ceramide metabolism in atherogenesis	2015	Experimental study	78 patients: 52 underwent carotid endarterectomy, and 26 underwent femoral endarterectomy	Analysis of ceramide, cholesterol, and beta-oxidation pathway to better comprehend the characteristics of atherosclerotic plaques
Vorkas et al. [51]	Metabolic phenotypes of carotid atherosclerotic plaques relate to stroke risk: An exploratory study	2016	Experimental study	Atherosclerotic plaque samples derived from 5 symptomatic patients and 5 asymptomatic patients who underwent CEA	Thanks to metabolomic technology, it is possible to distinguish between symptomatic and asymptomatic CS patients
Stegemann et al. [52]	Comparative lipidomics profiling of human atherosclerotic plaques	2011	Experimental study	26 patients with atherosclerotic disease	Lipidomic analysis of stable and unstable plaques of subjects with atherosclerotic disease
Wang et al. [53]	Gut microbiota, circulating inflammatory markers and metabolites, and carotid artery atherosclerosis in HIV infection	2023	Experimental study	Atherosclerotic plaque from 320 women with ^4^ HIV or at high risk of HIV	Association between a gut microbial metabolite, imidazole propionate, and CS
Cason et al. [54]	Plasma microbiome-modulated indole- and phenyl-derived metabolites associate with advanced atherosclerosis and postoperative outcomes	2018	Experimental, observational study	Plasma from 100 patients who underwent CEA and 22 healthy controls	Specific metabolites produced by gut microbes are connected with severe atherosclerosis and cardiac complications after interventions

^1^ Carotid stenosis; ^2^ carotid endoarterectomy; ^3^ non-esterified fatty acids; ^4^ human immunodeficiency virus.

**Table 10:** Principal features of the reviewed articles related to social issues and carotid stenosis.

AUTHORS	TITLE	YEAR OF PUBLICATION	RESEARCH DESIGN	SAMPLE CHARACTERISTICS	MAIN FINDING
Hsu et al. [55]	Gender, racial and ethnic disparities in index hospitalization operations for symptomatic carotid stenosis in Texas hospitals	2022	Experimental study	153,484 symptomatic patients with CS ^1^	Social factors that characterize carotid revascularization during the initial hospital stay for patients with symptomatic CS
Wu et al. [56]	Impact of neighborhood social disadvantage on carotid artery disease presentation, management, and discharge outcomes	2023	Experimental study	91,904 patients who underwent CEA ^2^, tfCAS ^3^, and TCAR ^4^	Social disadvantages like a low socioeconomic status determine severe CS and the need for TCAR
Baxi et al. [57]	Socioeconomic status as a predictor of post-operative mortality and outcomes in carotid artery stenting vs. carotid endarterectomy	2024	Experimental study	43,824 patients who underwent CEA and CAS ^5^	A low socioeconomic status is associated with stroke in patients who will undergo CEA and with mortality after the CEA intervention
Aber et al. [58]	Impact of carotid artery stenosis on quality of life: A systematic review	2019	Systematic review	None	Identification of 16 social themes as indicators of HRQoL ^6^ in patients with CS

^1^ Carotid stenosis; ^2^ carotid endoarterectomy; ^3^ transfemoral carotid artery stenting; ^4^ transcarotid artery revascularization; ^5^ carotid artery stenting; ^6^ health-related quality of life.
